# Molecular Mechanism of Vegetative Growth Advantage in Allotriploid *Populus*

**DOI:** 10.3390/ijms21020441

**Published:** 2020-01-10

**Authors:** Kang Du, Ting Liao, Yongyu Ren, Xining Geng, Xiangyang Kang

**Affiliations:** 1 Beijing Advanced Innovation Center for Breeding by Molecular Design, Beijing Forestry University, No. 35, Qinghua East Road, Haidian District, Beijing 100083, China; dukang@bjfu.edu.cn (K.D.); yongyuren@bjfu.edu.cn (Y.R.); gengxn@bjfu.edu.cn (X.G.); 2 Beijing Academy of Forestry and Pomology Sciences No. 12 A Rui Wang Fen, Fragrance Hills Haidian District, Beijing 100093, China; 3 Key Laboratory of Genetics and Breeding in Forest Trees and Ornamental Plants, Ministry of Education, Beijing Forestry University, No. 35, Qinghua East Road, Haidian District, Beijing 100083, China; 4 National Engineering Laboratory for Tree Breeding, College of Biological Sciences and Technology, Beijing Forestry University, No. 35, Qinghua East Road, Haidian District, Beijing 100083, China

**Keywords:** allotriploid, vegetative growth advantage, regulatory mechanism, dosage effect

## Abstract

Allotriploid poplar has a prominent vegetative growth advantage that impacts dramatically on lumber yield. The growth regulation is complex which involves abundant genes, metabolic and signaling pathways, while the information about the functional control process is very little. We used high-throughput sequencing and physiological index measurement to obtain a global overview of differences between allotriploid and diploid *Populus*. The genes related to plant growth advantage show a higher expression compared to diploid, and most of them are revolved around hormones, photosynthesis and product accumulation. Thus, allotriploid *Populus* showed more efficient photosynthesis, carbon fixation, sucrose and starch synthesis, and metabolism as well as augmented biosynthesis of auxin, cytokinin, and gibberellin. These data enable the connection of metabolic processes, signaling pathways, and specific gene activity, which will underpin the development of network models to elucidate the process of triploid *Populus* advantage growth.

## 1. Introduction

Polyploid plants often show prominent vegetative growth advantages [1,2]. These growth advantages include, but are not limited to, larger leaf area and cell size, more vigorous growth, and greater plant size [2,3]. For example, the leaf area and cell size were usually larger in *Arabidopsis* tetraploid plants than in diploid plants [2]. In *Zea mays*, triploid plants grew stronger than diploid plants [3,4], which was similar to Medicago tetraploids [5]. Among trees, *Populus* triploids manifest more vigorous growth and greater superiority than diploids. The biomass of triploids can reach 2–3 times that of diploids [6,7]. To maintain these growth advantages, polyploids demand more carbohydrates from greater assimilation of photosynthetic carbon [8,9,10,11]. According to previous reports, photosynthesis and chlorophyll content was higher in polyploids than diploids [2,9,12]. Research on different leaf canopy positions of *Populus* triploid populations suggested that triploid populations had a larger leaf area, higher chlorophyll content, and higher rates of photosynthesis compared to diploid populations [13]. However, the opposite can also be true. Tetraploid *Triticum aestivum* showed less photosynthesis than diploids [14]. 

The vegetative growth advantage in polyploids is certainly related to the increased genome size [15,16]. The question raised is what is the role of increased genome size in regulating genetics during vegetative growth? Differences in gene expression changes between polyploids and diploids have been documented in many plant species [17,18]. Previous research has reported that the expression of genes related to chlorophyll biosynthesis and sucrose and starch biosynthesis and metabolism was upregulated in *Arabidopsis* tetraploids compared with diploids. Growth advantages in *Arabidopsis* tetraploid plants were related to the circadian clock. During the day, the repressors *LHY* (LATE ELONGATED HYPOCOTYL) and *CCA1* (CIRCADIAN CLOCK ASSOCIATED1) were more inhibited in tetraploid plants than in the parents, which resulted in augmented photosynthesis and starch accumulation [2]. However, the influence of leaf position in a canopy gradient and the relationship between gene expression and miRNAs were not taken into account. Therefore, the molecular mechanisms of the vegetative growth advantage in polyploid plants require further investigation.

Physiological studies showed that two triploid populations (triploid-F and triploid-S) with different heterozygosity have significantly growth advantage than the diploid population. However, there are significant differences between triploid-F and triploid-S triploid populations in tree height, DBH (diameter at breast height) and leaf area [13]. This may be caused by the heterozygosity of parental transmission of gametes from different sources [19], so we selected two triploid populations for full research. Preliminary experiments founded that photosynthetic capacity maintained a high level from the 5th to the 10th leaf and showed stable senescence after the twenty-fifth leaf position. In this study, leaf canopy positions of two *Populus* allotriploid populations with different 2n gamete origins, triploid-F and triploid-S, and one diploid population from the same parents were used as the plant material [20]. We obtained the dynamic difference in the development of triploid and diploid leaves. The molecular mechanism of vegetative growth advantage in allotriploid plants was explored.

## 2. Results

### 2.1. Transcription Profile of DEGs (Differentially Expressed Genes) and RT-PCR (Real-Time Quantitative Polymerase Chain Reaction) Validation

**Figure 1:**
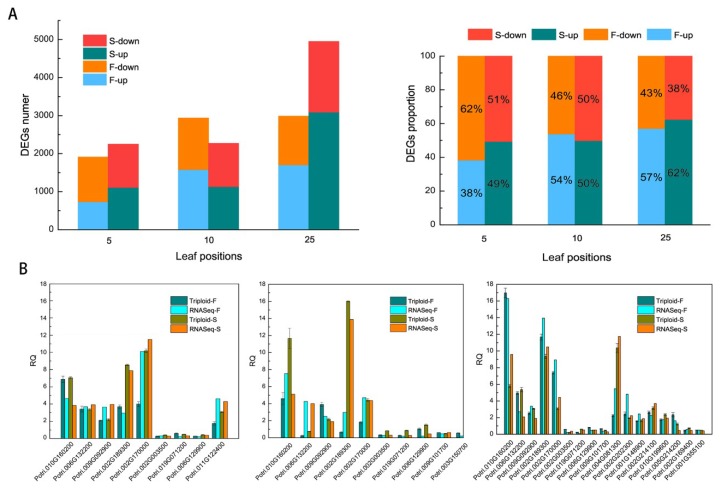
Transcription profile of differentially expresssed genes (DEGs) and real-time polymerase chain reaction (RT-PCR) validation. (**A**) The numbers and proportions of upregulated and downregulated genes in the 5th, 10th, and 25th leaves of triploid-F and triploid-S plants; (**B**) RT-PCR verification of randomly selected DEGs in the 5th, 10th, and 25th leaves of triploid-F and triploid-S plants.

Results showed 10,275 differentially expressed genes (DEGs) in total in triploid plants when diploids were used as the baseline. A total of 1911, 2938, and 2986 DEGs in triploid-F plants and 2248, 2272, and 4949 DEGs in triploid-S plants were identified in the 5th, 10th, and 25th leaves, respectively. The numbers of upregulated and downregulated genes in the 5th, 10th, and 25th leaves were shown in Figure 1A. There were fewer upregulated genes than downregulated genes in the 5th leaves of triploid-F plants and in the 5th and 10th leaves of triploid-S plants, whereas there were more upregulated genes than downregulated genes in the 10th and 25th leaves of triploid-F plants and in the 25th leaves of triploid-S plants (Figure 1A). A total of 36 DEGs on different leaf canopy positions were randomly selected for real-time polymerase chain reaction (RT-PCR) verification, and a comparison of the RT-PCR results and the RNA-seq data suggested that trends in changes in expression of most DEGs were in agreement (Figure 1B).

### 2.2. Chlorophyll Synthesis and Degradation

**Figure 2:**
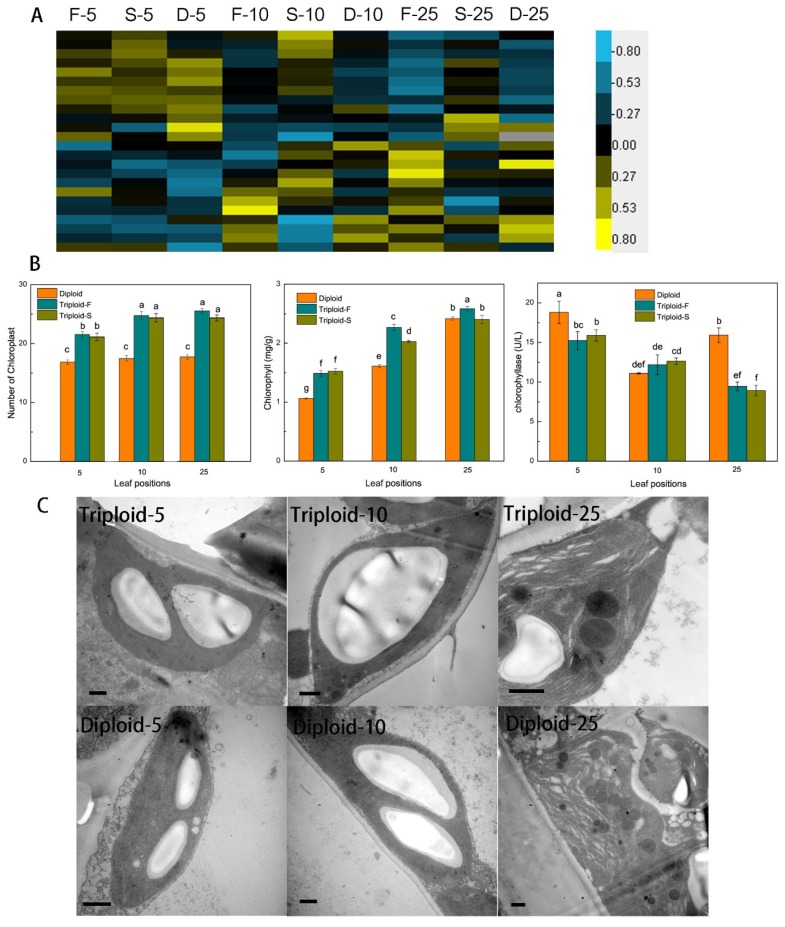
Chlorophyll synthesis and degradation in triploid-F, triploid-S and diploid plants. (**A**) Differentially expressed genes (DEGs) involved in chlorophyll synthesis and metabolism at different leaf canopy positions when triploid-F and triploid-S plants were compared to diploid plants; (**B**) The number of chloroplasts, chlorophyll content and chlorophyllase activity in triploid-F, triploid-S and diploid plants, different lowercase letters represent significant differences at *p* < 0.05; (**C**) Chloroplast ultrastructure in the 5th, 10th and 25th leaves in allotriploid and diploid plants. Bar = 10 μm.

A total of 24 genes involved in chlorophyll biosynthesis and metabolism showed a dosage effect and were differentially expressed (Figure 2A) in allotriploid plants compared to diploid plants. Seventeen genes were upregulated in the leaves at different positions of triploid-F and triploid-S plants, including *PORA* (protochlorophyllide oxidoreductase A), which can catalyze the synthesis of tetrapyrrol, prompting the conversion of protochlorophyll to chlorophyll. In addition, chloroplast and chlorophyll content was significantly greater in *Populus* allotriploids than diploids (Figure 2B). There were significant differences in numbers of chloroplasts between the 5th and 10th leaves of triploids, although no differences were found among the 5th, 10th, and 25th leaves, which indicates that chloroplasts were dividing between the 5th and 10th leaves of triploids. The number of mesophyll cell chloroplasts was 22.9 and 16.9 in triploids and diploids, respectively; therefore, the number of chloroplasts in triploids was about 1.35 times that in diploids. The chlorophyll content and chloroplast numbers of both triploid *Populus* was significantly different from that of diploids in the 5th and 10th leaves, but triploid-S plants did not differ from diploid plants in the 25th leaves (Figure 2B).

*CLH*, which is involved in chlorophyll degradation, was significantly downregulated in the 25th leaves of triploid-S plants. At the same time, chlorophyllase activity in the 5th and 25th leaves of triploid-S plants was significantly lower than in the same leaves of diploid plants (Figure 2B). The chloroplast ultrastructure of the 5th and 10th leaves did not differ between allotriploid and diploid plants. Chloroplasts were full of starch granules, starch granules in allotriploid plants were greater than in diploid plants, and the stroma lamella was complete (Figure 2C). However, compared to allotriploid plants, a wider range of osmiophilic granules were observed and the stroma lamella was more seriously damaged in the 25th leaves of diploid plants (Figure 2C), which indicated that the chloroplast aging rate was slower in allotriploids than in diploids. Therefore, *Populus* allotriploids showed a larger leaf area, a higher chlorophyll content, and a slower chloroplast aging rate compared to diploid plants.

### 2.3. Photoreaction and Carbon Fixation

**Figure 3:**
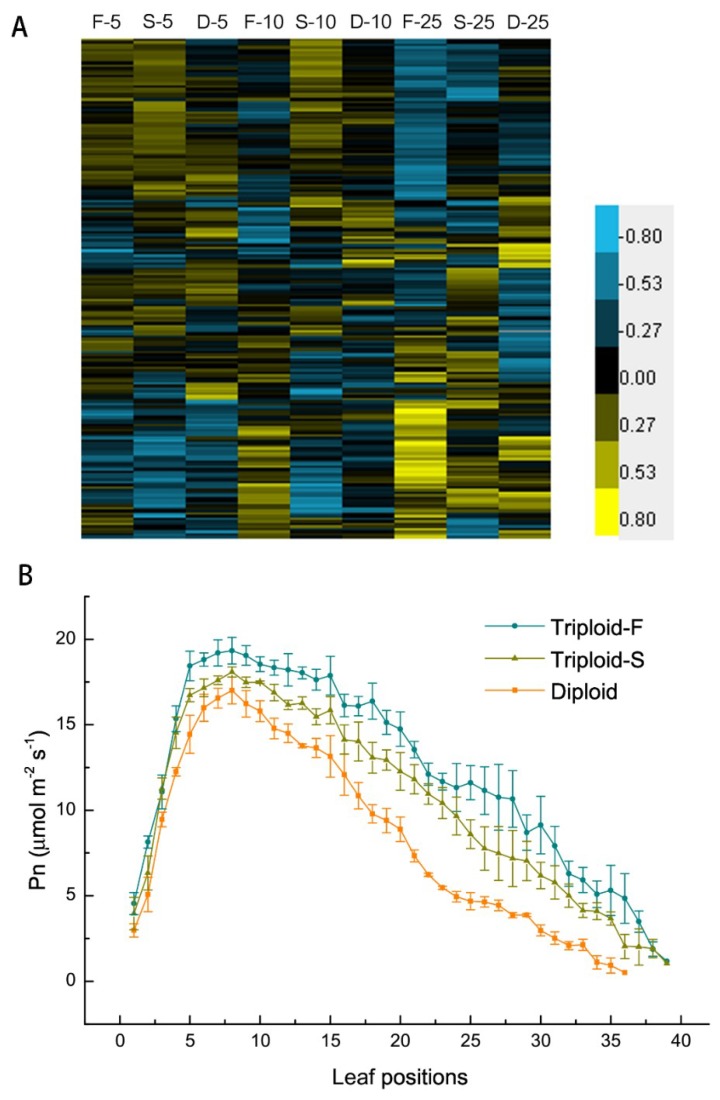
Photosynthesis in in triploid-F, triploid-S and diploid plants. (**A**) Differentially expressed genes (DEGs) involved in light reaction and carbon fixation on different leaf canopy positions when triploid-F and triploid-S plants were compared to diploid plants; (**B**) Net rates of photosynthesis on various leaf canopy positions in triploid-F, triploid-S plants and diploid plants.

There were 192 DEGs directly involved in or associated with light reaction and CO_2_ fixation in allotriploids compared to diploids. Of these DEGs, 135 were upregulated in two types of allotriploids and various leaf canopy positions, including the *LHC* (light harvesting complex) family members, which are involved in in light capture; two thylakoid membrane protein subunits, Psa and Psb family members; and key genes involved in the Calvin cycle, such as *GAPDH*, *FBP*, and *PRK* (Figure 3A). Allotriploid plants showed more efficient light capture, light reaction, and CO_2_ fixation compared to diploid plants. In addition, Pn was significantly greater in allotriploid plants than in diploid plants in various leaf canopy positions (Figure 3B), consistent with gene expression data. In the leaves below the 25th leaves, allotriploids still maintained a higher Pn, whereas the Pn of diploids decreased more quickly, which again indicates that chloroplast aging was slower in allotriploids than diploids.

### 2.4. Starch and Sucrose Synthesis and Metabolism

**Figure 4:**
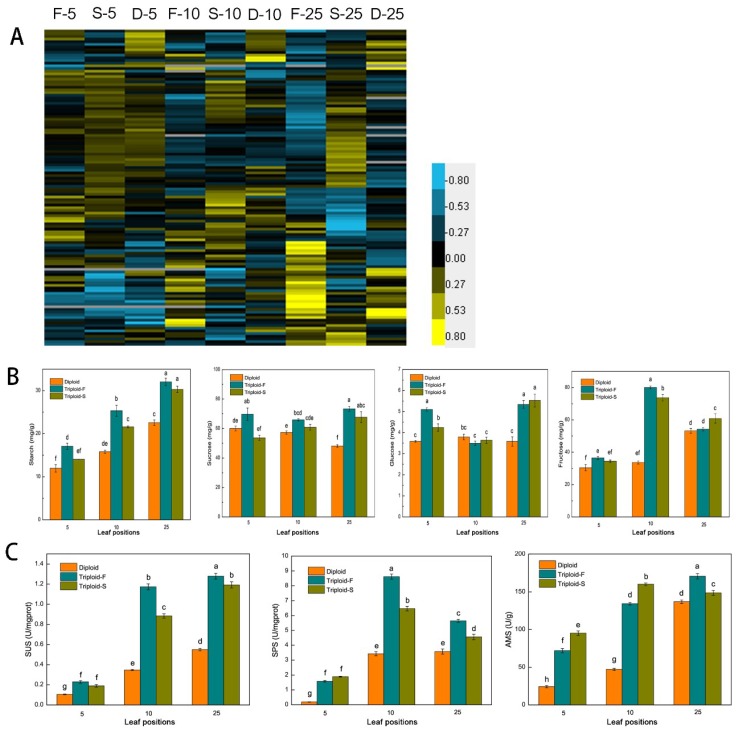
Starch and sucrose synthesis and metabolism in triploid-F, triploid-S and diploid plants. (**A**) Differentially expressed genes (DEGs) associated with sucrose and starch synthesis and metabolism on different leaf canopy positions when triploid-F and triploid-S plants were compared to diploid plants; (**B**) Starch, sucrose, glucose and fructose content on different leaf canopy positions when triploid-F and triploid-S plants were compared to diploid plants; (**C**) *SUS*, *SPS*, and *AMS* enzyme activity on different leaf canopy positions when triploid-F and triploid-S plants were compared to diploid plants. Different lowercase letters represent significant differences at *p* < 0.05.

A total of 118 genes associated with sucrose and starch metabolism showed differential expression patterns in allotriploids compared to diploids at different leaf canopy positions in *Populus*. Among them, 86 were upregulated in allotriploid plants, including genes involved in sucrose synthesis and degradation, such as *SUS* and *SPS*, and genes involved in starch synthesis and decomposition, such as *SS*, *ISA*, *AMS*, *DBE*, *BAM*, *AMY*, and *EMB* (Figure 4A). At the same time, sugar and starch contents were detected in leaves. Sucrose, glucose, and fructose contents were significantly higher in the 5th, 10th, and 25th leaves of the two types of allotriploid plants compared to the diploid plants (Figure 4B). The starch content increased with the increase in leaf position in both allotriploid and diploid plants, whereas there were no differences between the 25th leaves of triploid-F and triploid-S plants (Figure 4B). *SUS*, *SPS*, and *AMS* activity was significantly greater in allotriploid plants than diploid plants at the same leaf position (Figure 4C). In addition, enzyme activity was significantly greater in the 10th and 25th leaves than the 5th leaves (Figure 4C). Most genes related to sucrose and starch synthesis and metabolism in *Populus* allotriploids showed a dosage effect, which indicates that allotriploids had a greater ability to synthesize and transform sugar and starch.

### 2.5. Plant Hormone Synthesis and Signal Transduction

**Figure 5:**
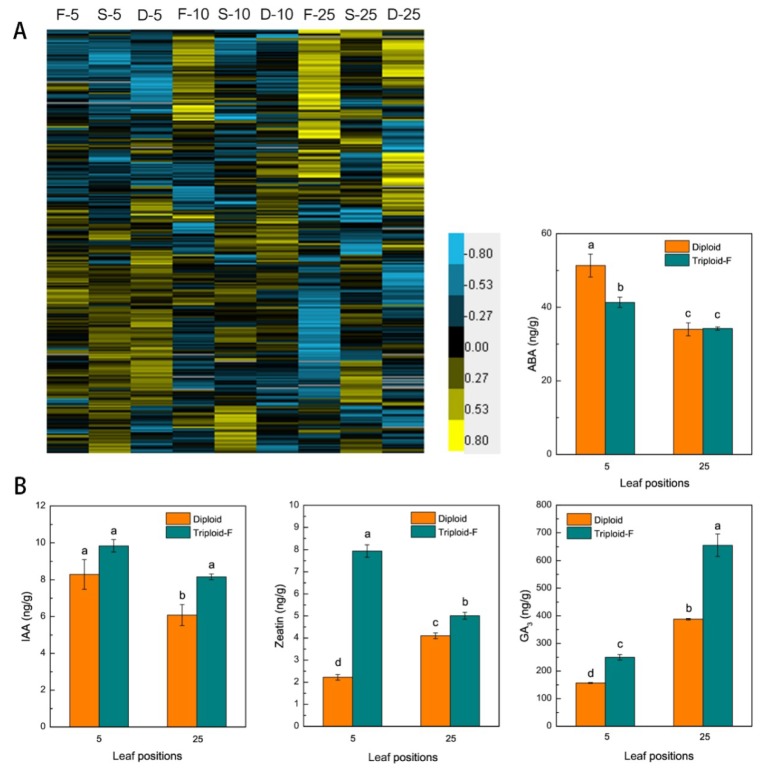
Hormone biosynthesis genes causing the upregulation of hormone in triploids. (**A**) Differentially expressed genes (DEGs) involved in plant hormone signal transduction on different leaf canopy positions when triploid-F and triploid-S plants were compared to diploid plants; (**B**) IAA, ZT, GA3 and ABA content in the 5th and 25th leaves of triploid-F and diploid plants. Different lowercase letters represent significant differences at *p* < 0.05.

In this study, 207 differentially expressed genes involved in plant hormone synthesis and signal transduction (Figure 5A). Including the auxin related genes (*ARFs*), cytokinins degradative genes (*CKX1*) and brassinosteroid signaling positive regulator (*BZR1*) were upregulated on various leaf canopy positions in allotriploids compared to diploids. The contents of IAA, GA3, zeatin (ZT), and ABA (abscisic acid) were measured in the 5th and 25th leaves of triploid-F and diploid plants. IAA, GA3, and ZT contents were higher in the 5th and 25th leaves, and ABA content lower in the 5th leaves, of triploid-F plants compared to diploid plants, whereas no differences were found between the 25th leaves of triploid-F and diploid plants (Figure 5B). Overall, the expression of genes involved in IAA, cytokinins (CTK), GAs, and brassinosteroids (BRs) synthesis and signal transduction was significantly higher in allotriploids than diploids. These hormones act alone and in concert to regulate vegetative growth advantages in *Populus* allotriploids.

### 2.6. Differentially Expressed miRNA Analysis

A total of 3181 miRNAs were detected by miRNA sequencing on the 5th leaves of allotriploid and diploid plants. Analysis of transcript levels by RT-PCR showed that miRNAs (R^2^ > 0.9, *p* <0.01) all displayed high correlation between the small RNA-seq and quantitative measurement suggesting that our high-throughput data were reliable (Figure S1). There were 12 (five upregulated, seven downregulated) and 10 (seven upregulated, three downregulated) differentially expressed miRNAs between triploid-F and triploid-S plants compared to diploid plants, respectively, and the fold changes ranged from 4.39 to 100 (Table 1). The target gene expression and function of miRNAs were analyzed using transcriptome data of the same materials. A total of 22 differentially expressed miRNAs could be applied to 227 target gene loci of 155 target genes, and one miRNA applied to 10.32 loci on average. Functional annotation of target genes showed that they were involved in stress defense, lignin synthesis, programmed cell death, response to gibberellins stimulation, regulation of secondary cell-wall synthesis, growth, cell division, and sucrose metabolism processes (Table S1). A total of 15 genes in the 22 target genes were differentially expressed when combined with the transcriptome data, and 14 pairs of miRNA and mRNA showed a negative regulating relationship (Table 2). Target genes directly related to plant vegetative growth characteristics were not found.

To verify the differences in miRNAs regulating the vegetative growth advantage between allotriploid and diploid plants, we used DEGs in the 5th leaves of allotriploid and diploid plants related to chloroplast decomposition, photoreaction, carbon fixation, sucrose, starch synthesis and decomposition, plant hormone synthesis, and signal transduction as target genes and reversed them to identify their corresponding miRNAs. A total of 123 DEGs from triploid-F plants and 113 DEGs from triploid-S plants were involved in or associated with bioprocesses related to vegetative growth. These 123 and 113 vegetative genes were regulated by 54 and 46 miRNAs (Tables S2 and S3), respectively. However, expression of these miRNAs did not differ significantly between allotriploid and diploid plants.

## 3. Discussion

### 3.1. Temporal Differences in Gene Expression Patterns in Populus Allotriploid Plants

**Figure 6:**
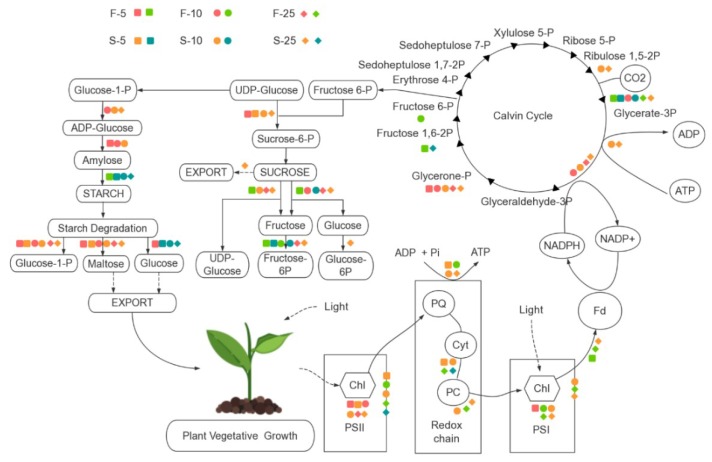
DEGs involved in metabolism pathway in the leaves of triploid-F and triploid-S plants. Warm and cool colors represent upregulated and downregulated expression, respectively.

**Figure 7:**
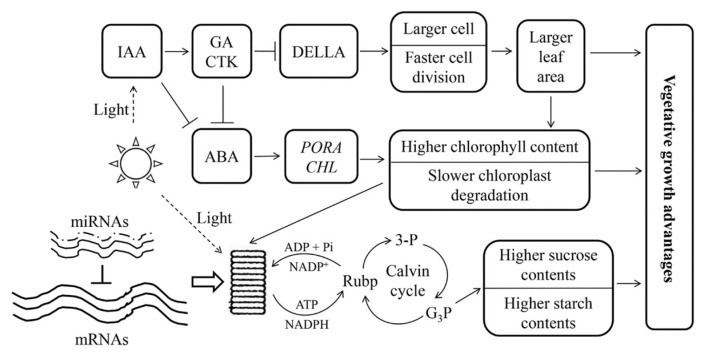
A model of the vegetative growth advantages of *Populus* allotriploid. Arrows and short-term indicate promotion and inhibition, respectively. The solid lines of mRNAs represent the dosage effect in *Populus* allotriploid, and the dashed lines of miRNAs represent no dosage effect in *Populus* allotriploid. 3-P: triphosphoglycerate; G_3_P: triglyceride phosphate.

In this study, the same cultivation environment and sampling time were used for both triploid and diploid plants. When the diploid was used as a control, the DEGs from triploid-F and triploid-S were different, and so were the DEG sets from various leaf canopy positions in triploids (Figure S2). Only 6.3–47.2% of genes showed a dosage effect on pathways directly related to vegetative growth (Table S4). The DEGs related to chlorophyll synthesis, photosynthesis, and sucrose and starch synthesis and metabolism in different leaves of triploid plants showed several discrepancies (Figure 6). For example, *LHCA2* and *LHCA6* showed upregulated expression in the 5th leaves and no difference in the 10th leaves, and *LHCA2* had downregulated expression in the 25th leaves of triploid-F plants compared to diploid plants. However, *LHC* showed no difference in the 5th leaves and upregulated expression in the 10th leaves, and *LHCA2* showed downregulated expression in the 25th leaves of triploid-S plants compared to diploid plants. These differences in gene expression dosage indicated that gene transcription in allotriploid plants had some temporal aspects and patterns. The dosage effect genes that generated the enzyme protein in the metabolic pathway depended on the product formed in the previous stage [21]. The product of the dosage effect genes was the substrate of downstream genes in the next stage of the metabolic pathway [22,23]. The temporal difference in gene expression patterns may be a kind of adaptive behavior for budgeting energy and making full use of the cellular apparatuses in the metabolic process in allotriploid *Populus* (Figure 7).

### 3.2. Gene Expression Differences between Populus Allotriploids and Diploids

The contribution of photosynthesis to plant vegetative growth is largely determined by leaf area, chlorophyll content per leaf area, and chloroplast life span [12,14]. A greater number of chloroplasts and higher chlorophyll content in allotriploids might be a result of the significantly greater expression of protochlorophyllide oxidoreductase (POR) in *Populus* allotriploids compared to diploids. POR catalyzed the synthesis of tetrapyrrol and promoted protochlorophyllide to make chlorophyll [14,20]. Similar results were reported in *Arabidopsis* tetraploids. The leaf chlorophyll content was higher in *Arabidopsis* tetraploids than in diploids because of upregulated expression of *PORA* and *PORB* in *Arabidopsis* tetraploids [2]. However, the degradation of chlorophyll and chloroplasts led to leaf senescence directly [24,25]. In the present study, the activity of chlorophyllase was weaker in the 25th leaves of triploid-S plants than in diploid plants, and electron microscopy ultrastructure observation showed that degradation of chloroplasts was slower in allotriploid plants. These findings might be related to the chlorophyllase gene (*CLH*), which regulates the degradation of chlorophyll and was downregulated in the 25th leaves of triploid-S plants compared to diploid plants.

As the main light-capture protein complexes, *LHCII* controlled light quantum divergence and transfer [26]. In the present study, most of the *LHC* gene family members showed upregulated expression in allotriploid plants compared to diploid plants, which indicates that light capture was more efficient than in diploid plants. *STN7* was an important gene in the process of *LHCII* phosphorylation and controlled the expression of the photosynthesis gene [27]. Palareti reported that the phosphorylation signal was disturbed in the absence of *STN7* mutant materials in *Arabidopsis* and that efficiency of photosynthesis was altered [28]. *STN7* showed upregulated expression in the present triploid plants compared to diploid plants, which indicates that the triploids had a more efficient phosphorylation process compared to the diploids. PSI and PSII were the subunit protein complexes that embedded in the thylakoid membrane (mainly including the Psa and Psb gene families), driving the light reaction [29]. Most members of the Psa and Psb gene families, including PsaK, PsaF, PsbP, and PsbQ, were also upregulated in triploid plants compared to diploid plants, which indicates that the light reaction was more efficient in triploids than in diploids.

Starch was the final product of photosynthesis, and more efficient photosynthesis led to the accumulation of the photosynthetic product. To maintain the material balance in cells, sugars and starches need more active catabolism and transfer processes. SUS, SPS, SS, AMS, BAM, and PHS were the main enzymes involved in the synthesis and metabolism of sugar and starch [30]. Enhancing SPS activity increased sucrose content in *A. thaliana* [31]. Ni found that tetraploids had higher sucrose and starch content and a greater ability to synthesize and decompose starch in *A. thaliana*. BAM, the PHS related to starch decomposition that upregulated expression in *A. thaliana* tetraploids during the day, resulted in a growth advantage in *A. thaliana* tetraploids compared to diploids [2]. Allotriploid plants showed higher sugar and starch content, and the enzyme activity of SUS, SPS, and AMS was comparable to that of the diploid in 5th, 10th, and 25th leaves. Moreover, most genes related to enzyme synthesis showed upregulated expression patterns in different leaf positions of *Populus* allotriploids, indicating vigorous synthesis and decomposition capacity, and efficient sugar transport processes could ensure more energy for growth and development in *Populus* allotriploids [32,33,34].

Higher levels of IAA, GAs, CTK, and BRs could promote protein synthesis, cell division, and differentiation; maintain the high growth advantage; and prevent leaf aging [35]. GAs compounded mainly through GA20ox, Ga3ox family members [36]. BRs promoted the photosynthesis and growth rate and coordinated the interaction with IAA [37]. A transcription factor, *BZR1*, involved in the BRs signal transduction [38]. The genes *GA20ox2*, *GA20ox4*, *GA3ox1*, *BZR*, and *BRI*, which were related to the synthesis and response of ZT, BRs, IAA, and GAs, were significantly upregulated in allotriploid plants compared to diploid plants. The result was that IAA, ZT, and GA3 content was higher in allotriploid plants compared to diploid plants. Therefore, triploids had a greater ability to undergo cell division than diploids (Figure 7).

### 3.3. Expression of Negative Regulatory Factors in Populus Allotriploids

As a type of negative regulatory factors, miRNAs inhibited the expression of their target genes usually through perfect or nearly perfect complementation with target gene sequences [39]. In the present study, 10–12 differentially expressed miRNAs were detected in 3181 miRNAs from triploid-F and triploid-S plants compared to diploid plants, respectively, and failed to find target genes significantly associated with vegetative growth. The expression of miRNAs related to chlorophyll synthesis and decomposition, photosynthesis, and sugar and starch synthesis and decomposition did not differ between allotriploid and diploid plants, which demonstrated that the expression of miRNAs related to vegetative growth in the leaves of *Populus* allotriploids showed no dosage effect. Similarly, 2273 detected miRNAs showing no significant differences in expression between allotriploid and diploid plants were reported by Suo on the terminal bud of the same origin used in this study [19].

The circadian clock had a regulatory role in polyploid growth advantage. It impacted oscillator or signal systems in higher plants, but the molecular regulating mechanisms are still unclear. The circadian clock regulated gene expression by regulating circadian rhythms and significantly affected plant growth and development [40]. Ni showed that *CCA1* and *LHY*, which are upstream of transcription factors, were more inhibited during the day in *Arabidopsis* allotriploids than in diploids [2]. This allowed for the expression of regulon *TOC1* and *GI*, and then downstream genes were upregulated, including genes related to chlorophyll synthesis, photosynthesis, and sucrose and starch metabolism. We believed that there was a connection between the circadian clock and vegetative growth in *Arabidopsis* and that polyploid plants could better use the daytime light. In the present study, expression of *LHY* in the 5th leaves at 9:00 am in *Populus* allotriploids at various leaf canopy positions did not differ from that of diploids (Table S5). Expression of *TOC1* and *GI* did not differ between allotriploid and diploid plants. There were no homologous genes of *A. thaliana CCA1* in *Populus* leaves, which suggest inhibition of *CCA1* might not work in *Populus* allotriploids. Our results indicated that different regulatory mechanisms might exist in different species in circadian clock gene expression in polyploid plants.

## 4. Materials and Methods

### 4.1. Plant Materials

*Populus* allotriploid populations (2n = 3× = 57) were generated from the hybridization of induced 2n female gametes of *Populus pseudosimonii* × *P. nigra* ‘Zheyin3#’ and *P. × beijingensis*. The allotriploid plants divided into first-division restitution types and second-division restitution 2n megaspore types according to Dong’s methods [20,41]. The allotriploid population that originated from first-division restitution 2n female gametes was named “triploid-F”, and the population derived from second-division restitution 2n female gametes was named “triploid-S”. The diploid population originating from the same parents was used as a baseline control. The origin of materials used in the present research are shown in Figure S3. Nine seedings were randomly selected in each population and cultivated in greenhouse from previous results [13]. Three position leaves (5th, 10th, 25th leaf from stem apex) were collected from healthy tissue seedlings grown for 3 months. Equal amounts of the leaves from three diploid (or alltriploid) same position were mixed as one sample. Three biological replicates were used for each genotype.

### 4.2. Transcriptome Analysis

The 5th, 10th, and 25th leaf samples used for genome-wide transcriptome sequencing in the triploid-F, triploid-S, and diploid plants were harvested and placed into cryopreserved tubes and then quickly frozen in liquid nitrogen. A TRIzol reagent kit (Invitrogen, Carlsbad, CA, USA) was used to extract the total RNA in leaf samples, and an RNase-Free DNaseSet (Qiagen China, Shanghai, China, https://www.qiagen.com/cn/) was used to purify the RNA. To detect the integrity of the RNA, we performed agarose gel electrophoresis, and a NanoDrop 2000 biological analyzer (Thermo Fisher Scientific Inc., Wilmington, DE, USA) was used to detect the concentration of RNA. After this quality control check (1.6 ≤ 28 s/18 s ≤ 2.0), high-quality RNA was used for subsequent sequencing. We built mixed gene pools using a mixture of three candidate genotypes in the 5th, 10th, and 25th leaves of triploid-F, triploid-S, and diploid plants, respectively, with three biological duplications. Following Wang et al., [42], an Ion total RNA-Seq Kit v2 (Life Technologies, Santa Clara, CA, USA) was used to build the cDNA libraries at different leaf positions. Transcriptome sequencing of leaf samples was performed on the Ion Proton platform (Life Technologies) by Shanghai Novelbio Biological Technology Co., Ltd. (Shanghai, China, http://www.novelbio.com/). Original reads less than 50 bp were filtered out, and the rest were mapped to the genome of *Populus trichocarpa* using MapSplice [43]. The abundance of transcription was shown by reads per kilobase per million mapped reads (RPKM). DESeq R package was applied for differential gene expression analysis [44]. The resulting *p*-values were adjusted by Benjamini and Hochberg’ approach for controlling false discovery rate [45]. Genes with adjusted *p*-value < 0.05 and fold changes |log_2_(FC)| > 1 were considered as differentially expressed. Information on gene annotation was acquired from the NCBI (http://www.ncbi.nlm.nih.gov/), JGI (http://jgi.doe.gov/), PopGenie (http://popgenie.org/), Tair (http://www.arabidopsis.org/), and KEGG (http://www.kegg.jp/) databases.

### 4.3. RT-PCR Validation of Differentially Expressed Genes

A FastQuant RT kit (with gDNase; Tiangen Biotech Co., Ltd., Beijing, China) was used to perform the reverse transcription reaction. Real-time polymerase chain reaction (RT-PCR) was accomplished using a SuperReal PreMix Plus (SYBR Green, Beijing, China) kit (Tiangen Biotech, Beijing, China) using an Applied Biosystems 7500 Fast instrument (AB Ltd., Lincoln, NE, USA). The RT-PCR master mix included 10 µL 2 × SuperReal PreMix Plus, 0.6 µL forward primer, 0.6 µL reverse primer, 2 µL cDNA template, 0.4 µL 50 × ROX Reference Dye, and 6.4 µL RNase-free ddH2O. RT-PCR was performed using 39 cycles of the following conditions: 95 °C for 15 min for pre-degeneration, 95 °C for 10 s for degeneration, 58 °C for 30 s for annealing, 72 °C for 30 s for extension. Afterward the samples were heated to 95 °C for 15 s and then 60 °C for 1 min for dissolution curve analysis. Four technical replicates and three biological replicates were used for each sample and randomly selected gene. The sequences of primers used in the present study were designed using Primer3Plus (http://www.primer3plus.com/) and were listed in Table S6. Actin (accession number: EF145577) was chosen as the reference gene, and the −2^−ΔΔCt^ method was used to calculate gene expression.

### 4.4. Measurement of Photosynthesis

Pn was determined on the first fully expanded leaf to the last leaf from the top of the stem in the three highest Pn genotypes using the LI-6400-02B portable photosynthesis system (LiCor, Lincoln, NE, USA). Measurements were taken on sunny days at 08:30–11:30 a.m., with maintenance of the photosynthetic photon flux density at 1400 µmol (photon) m^−2^·s^−1^ and a CO_2_ concentration of 400 µmol mol^−1^, relative air humidity of 60–65%, and flow of 500 µmol·mol^−1^.

### 4.5. Measurement of Chlorophyll, Starch and Sugar Content

Chlorophyll, starch, and sugar content were measured in the 5th, 10th, and 25th leaves of the three selected candidate genotypes using three biological replicates. Chlorophyll content was determined as described by Vernon & Seely [46] with slight modifications. A total of 1 g fresh leaf tissue was ground into a homogenate with 5 mL 95% ethanol with a small quantity of quartz sand and calcium carbonate powder. Then the homogenate was ground in an additional 5 mL 95% ethanol. After standing for 3 min, the extract was filtered to a 50 mL brown volumetric flask, diluted with 95% ethanol to 50 mL, and then mixed. The absorbance of the filtrate was measured at 645 and 663 nm using a spectrophotometer (Ultrospec 6300 Pro, Cambridge, UK). Chlorophyll content was calculated as follows: chlorophyll content (mg·g^−1^) = 8.02 × A663 + 20.20 × A645. 

Starch and sugar contents were also measured in the 5th, 10th, and 25th leaves of triploid-F, triploid-S, and diploid plants. Then 10% of the crude extract was homogenized with 0.1 g powdered fresh leaf tissue in 0.9 mL 1× phosphate buffer (pH 7.2). Starch, sucrose, fructose, and glucose content was determined using EnzyChrom^TM^ assay kits (BioAssay Systems Co., Hayward, CA, USA, https://www.bioassaysys.com/) and expressed as milligrams of substance per gram of fresh leaf.

### 4.6. Enzyme Assay

Similar to the determination of starch and sugar content, 10% of the enzymatic crude extract of the 5th, 10th, and 25th leaves of triploid-F, triploid-S, and diploid plants were prepared. Sucrose synthase (SUS), sucrose phosphate synthase (SPS), and amylase (AMS) activities were assayed using enzyme-linked immunosorbent assay (ELISA) kits (Comin Commodities Co., Ltd., Zhejiang, China, http://www.comin.biz/index.html). Chlorophyllase activity was determined using a chlorophyllase ELISA kit (GeneTex, Inc., Irvine, CA, USA, http://www.genetex.com/).

### 4.7. Plant Materials

The ultrastructure of chloroplasts in the 5th, 10th, and 25th leaves was manufactured using the method of Otegui et al. [47] with minor modifications. Leaf samples were sectioned with a razor blade into small pieces of 2 × 2 mm and rapidly infiltrated with 2.5% glutaraldehyde for 4 h. After being dipped in 1× phosphate buffer three times for 30 min each time, leaves were infiltrated into 1% OsO_4_ for 2 h, then washed with 1× phosphate buffer for 30 min each time. The leaves were dehydrated for 20 min in 30%, 50%, 70%, and 90% acetone, followed by two washes with 100% acetone. After dehydration, the leaves were infiltrated twice for 12 h with 100% acetone mixed with 25%, 50%, 75%, or 100% resin. Afterward, leaves were placed into an embedding plate with fresh resin, and polymerization was carried out at 60 °C for 12 h, followed by cooling at room temperature. Aggregation blocks were cut into slices of 1 µm thickness using an ultra-thin slicing machine (Leica EM UC7, Berlin, Germany). Sections were stained with 2% uranium acetate in 70% methanol for 10 min followed by Reynold’s lead citrate (2.6% lead nitrate and 3.5% sodium citrate, pH 12) for 5 min. The chloroplast ultrastructure was observed through a JEM-1010 electron microscope (Jeol Ltd., Tokyo, Japan).

### 4.8. Determination of Plant Endogenous Hormone Content

Plant endogenous hormone content, including gibberellic acid (GA3), auxin (IAA), zeatin (ZT), and abscisic acid (ABA), was determined in the 5th and 25th leaves of triploid-F and diploid plants using the high-performance liquid chromatography–mass spectrometry (LC–MS) method as described in Pan et al. [48]. Leaves were harvested, immediately frozen in liquid nitrogen, and then transferred to a −80 °C refrigerator. The leaf tissue of each sample was ground to powder in liquid nitrogen, and 50 mg powder was transferred into a 2 mL centrifuge tube, followed by the addition of 500 µL extraction agent (2-isopropyl alcohol:water:hydrochloric acid = 2:1:0.002). The internal standard solution of plant hormone contained 2 ng/µL GA_3_ 5 µL, 2 ng/µL IAA 5 µL, 0.25 ng/µL ABA 15 µL, and 0.25 ng/µL ZT 10 µL. Then we placed the centrifuge tubes on a rolling bed at 4 °C, 100× *g*, for 30 min before adding 1 mL methylene chloride to each tube. The tubes were shaken for an additional 30 min, and the mixed solution was centrifuged at 4 °C, 12,000× *g*, for 10 min. The lower fraction was transferred to a clean centrifuge tube and dried with nitrogen gas. Then, 200 µL methanol was added to dissolve the leaf sample. A 10 µL sample was used for LC–MS using an AB Sciex QTRAP 5500 LC system (AB Sciex Pte., Ltd., South Burlington, Vermont, USA), with acetonitrile and 0.4% triethylamine solution (pH 3.5) used as the mobile phase. We used biological replicates (samples) for each genotype, and each replicate was performed with three technical replicates.

### 4.9. miRNAs Analysis

Samples of the 5th leaves of triploid-F, triploid-S, and diploid plants were taken at 9:00 a.m. and frozen in liquid nitrogen. An RNA 6000 Nano Assay Kit and 2100 Bioanalyzer RNA Nanochip (Agilent) were used to control the quality of purity and concentration of RNA, respectively (RIN ≥ 8.0), and a miRNeasy Mini Kit (Qiagen, shanghai, China) was used to purify the RNA. Similar to transcriptome sequencing, miRNA sequencing was completed on the Ion Proton platform (Life Technologies) by Shanghai Novelbio Biological Technology after the cDNA libraries were built using a mixture of the three candidate genotypes of triploid-F, triploid-S, and diploid plants, respectively. High-quality reads after filtering were mapped to the miRNA database of *Populus trichocarpa* (Release 21.0, http://www.mirbase.org/) using BWA (v0.7.5a). To obtain the new candidate miRNA precursor, unmapped reads were further mapped to the genome of *P. trichocarpa*. To obtain more homologous miRNAs, unmapped reads were mapped one by one onto the miRNA databases of *Arabidopsis thaliana*, *Glycine max*, *Zea mays*, and *Oryza sativa*. The target genes of miRNAs were predicted using psRNATarget (plantgrn.noble.org/psRNATarget).

## 5. Conclusions

In summary, different vegetative growth advantages in polyploid plants formed as a result of different regulatory mechanisms. A new molecular regulatory mechanism of vegetative growth advantage in allotriploid plants was discovered in the present study. The expression of genes related to vegetative growth had a dosage effect, whereas the expression of related miRNAs showed no dosage effect in *Populus* allotriploids. Gene transcription and dosage expression were time-dependent. In addition, gene expression levels inhibited by miRNAs were lower, leading to less negative control in target genes by miRNAs in *Populus* allotriploids compared to diploids. Furthermore, the leaf area was bigger and the chloroplast aging rate was slower in allotriploids than in diploids, leading to augmented effects of light reaction; carbon fixation; sucrose and starch synthesis and metabolism; and synthesis of IAA, CTK, and GA3 in allotriploids compared to diploids. All of these findings together promote a vegetative growth advantage in *Populus* allotriploids .

## Figures and Tables

**Table 1:** Differentially expressed microRNAs in allotriploid and diploid *Populus*.

Group	AccID	Sequence	FC	Style	*p*-Value	FDR
**F5 vs. D5**	chr5_13131_mature	acagaatcagagttcacaggcct	7.76	up	3.24 × 10^−5^	1.51 × 10^−2^
	chr17_37159_mature	gcatcaatttatcttagtagcca	6.21	up	4.08 × 10^−5^	1.51 × 10^−2^
	chr7_17886_mature	gtggatcatgtattttgatgg	5.46	up	1.09 × 10^−4^	2.14 × 10^−2^
	ptc-miR169t	gagccaagaatgacttgccgg	0.07	down	2.23 × 10^−8^	4.38 × 10^−5^
	chr17_37159_star	tgcaatgtagggaatagatgtg	9.23	up	4.39 × 10^−6^	4.32 × 10^−3^
	ptc-miR395k	ctgaagtgtttgggggaactc	0.18	down	7.72 × 10^−5^	1.90 × 10^−2^
	ptc-miR395j	ctgaagtgtttgggggaactc	0.20	down	1.72 × 10^−4^	3.07 × 10^−2^
	ptc-miR395h	ctgaagtgtttgggggaactc	0.17	down	4.09 × 10^−5^	1.51 × 10^−2^
	ptc-miR395f	ctgaagtgtttgggggaactc	0.17	down	4.62 × 10^−5^	1.51 × 10^−2^
	ptc-miR395d	ctgaagtgtttgggggaactc	0.19	down	8.91 × 10^−5^	1.95 × 10^−2^
	ptc-miR395b	ctgaagtgtttgggggaactc	0.17	down	5.45 × 10^−5^	1.53 × 10^−2^
	chr13_29473_mature	ttttggtcggtatgtcttgc	4.39	up	2.45 × 10^−4^	4.02 × 10^−2^
**S5 vs. D5**	chr8_20880_mature	tacacctgacatttatggcat	6.42	up	6.04 × 10^−5^	1.70 × 10^−2^
	chr5_13131_mature	acagaatcagagttcacaggcct	10.70	up	3.07 × 10^−6^	3.03 × 10^−3^
	chr17_37159_mature	gcatcaatttatcttagtagcca	6.90	up	1.48 × 10^−5^	7.29 × 10^−3^
	chr7_17723_mature	tatgacatcttgatatata	0.01	down	3.43 × 10^−18^	6.77 × 10^−15^
	chr7_17886_mature	gtggatcatgtattttgatgg	5.20	up	1.18 × 10^−4^	2.91 × 10^−2^
	ptc-miR169t	gagccaagaatgacttgccgg	0.15	down	3.65 × 10^−5^	1.44 × 10^−2^
	chr17_37159_star	tgcaatgtagggaatagatgtg	7.81	up	5.05 × 10^−5^	1.66 × 10^−2^
	chr3_8559_star	tggcattttctccttttgagttt	0.14	down	1.75 × 10^−4^	3.84 × 10^−2^
	chr13_29473_mature	ttttggtcggtatgtcttgc	6.84	up	5.68 × 10^−6^	3.73 × 10^−3^
	chr1_2404_mature	acacactgcatctaaaaacta	5.21	up	2.00 × 10^−4^	3.94 × 10^−2^

FC: fold change; FDR: false discovery rate; D5, F5, S5 represent diploid, triploid-F, and triploid-S plants in the 5th leaves in the morning, respectively.

**Table 2:** Targeted negative regulation relationship in miRNA-mRNA.

Group	miRNA_ID	miRNA_FC	Style	FDR	Target Gene_ID	Gene_FC	Style	FDR	Tair	Symbol	Description
**F5 vs. D5**	chr17_37159_star	9.23	up	4.32 × 10^−3^	Potri.001G375700	0.43	down	2.44 × 10^−2^	AT3G15050	IQD10	IQ-domain 10
	ptc-miR169t	0.07	down	4.38 × 10^−5^	Potri.006G053500	2.87	up	4.10 × 10^−3^	AT3G14020	NF-YA6	nuclear factor Y, subunit A6
	ptc-miR395b	0.17	down	1.53 × 10^−2^	Potri.010G081200	1.53	up	2.96 × 10^−2^	AT3G22890	APS1	ATP sulfurylase 1
	ptc-miR395d	0.19	down	1.95 × 10^−2^	Potri.010G081200	1.53	up	2.96 × 10^−2^	AT3G22890	APS1	ATP sulfurylase 1
	ptc-miR395f	0.17	down	1.51 × 10^−2^	Potri.010G081200	1.53	up	2.96 × 10^−2^	AT3G22890	APS1	ATP sulfurylase 1
	ptc-miR395h	0.17	down	1.51 × 10^−2^	Potri.010G081200	1.53	up	2.96 × 10^−2^	AT3G22890	APS1	ATP sulfurylase 1
	ptc-miR395j	0.20	down	3.07 × 10^−2^	Potri.010G081200	1.53	up	2.96 × 10^−2^	AT3G22890	APS1	ATP sulfurylase 1
	ptc-miR395k	0.18	down	1.90 × 10^−2^	Potri.010G081200	1.53	up	2.96 × 10^−2^	AT3G22890	APS1	ATP sulfurylase 1
	chr5_13131_mature	7.76	up	1.51 × 10^−2^	Potri.012G083100	0.41	down	2.78 × 10^−2^	AT4G10650	AT4G10650	P-loop containing nucleoside triphosphate hydrolases superfamily protein
**S5 vs. D5**	chr7_17886_mature	5.46	up	2.14 × 10^−2^	Potri.014G185300	0.29	down	4.25 × 10^−2^	AT5G42710	AT5G42710	unknown protein
	chr17_37159_star	7.81	up	1.66 × 10^−2^	Potri.007G053400	0.11	down	3.71 × 10^−5^	AT5G67400	RHS19	root hair specific 19
	chr17_37159_star	7.81	up	1.66 × 10^−2^	Potri.010G239300	0.63	down	3.89 × 10^−2^	AT4G39200	AT4G39200	Ribosomal protein S25 family protein
	chr3_8559_star	0.14	down	3.84 × 10^−2^	Potri.005G119700	4.96	up	2.07 × 10^−2^	AT2G18180	AT2G18180	Sec14p-like phosphatidylinositol transfer family protein
	chr5_13131_mature	10.70	up	3.03 × 10^−3^	Potri.012G083100	0.34	down	4.87 × 10^−3^	AT4G10650	AT4G10650	P-loop containing nucleoside triphosphate hydrolases superfamily protein

FC: fold change; FDR: false discovery rate; D5, F5, S5 represent diploid, triploid-F, and triploid-S plants in the 5th leaves in the morning, respectively. ptc- means the miRNAs detecked in *Populus trichocarpa* miRNA database. Chl_ means the novel miRNA for *Populus cathayan*.

## Supplementary Materials

Supplementary materials can be found at https://www.mdpi.com/1422-0067/21/2/441/s1.

## Abbreviations

DEGs	Differentially Expressed Genes
CTK	Cytokinins
BRs	Brassinosteroid
